# Hairy Cell Leukemia Following Acute Myeloid Leukemia, Concomitant or Secondary?

**DOI:** 10.34172/aim.28846

**Published:** 2024-11-01

**Authors:** Zhang Bingyao, Fu Zhaoqiang, Zhang Xuxi, Yang Qian, Qin Youwen

**Affiliations:** ^1^Department of Clinical Laboratory, Shanghai Zhaxin Traditional Chinese and Western Medicine Hospital, Shanghai, China

**Keywords:** Acute myeloid leukemia, Allogeneic hematopoietic stem cell transplantation, Hairy cell leukemia

## Abstract

A 62-year-old man diagnosed with acute myeloid leukemia (AML) showed limited responses to two courses of azacitidine (AZA)+Venetoclax (VEN) therapy. Twenty days after being transferred to our hospital, flow cytometry with broad antigen coverage and mutation analysis confirmed the presence of a second malignancy, hairy cell leukemia (HCL). Following haploidentical combined umbilical cord blood transplantation, the patient achieved complete remission (CR) for both AML and HCL. This CR has been maintained for the past 14 months. Patients with dual hematologic malignancies may not respond well to conventional therapy regimens. Early initiation of hematopoietic stem cell transplantation is beneficial for improving prognosis and extending overall survival.

## Introduction

 Several studies have documented the incidence of acute myeloid leukemia (AML) in patients with hairy cell leukemia (HCL).^1-4^ The heightened risk of secondary cancers in these cases has been attributed to either the immune dysfunction observed in HCL patients or as a consequence of the treatment for HCL. While the occurrence of HCL as a second malignancy following AML therapy is extremely rare and has not been previously reported, concomitant AML and HCL were reported in two patients.^5,6^ In this report, we present the case of a patient with AML who developed HCL shortly after undergoing treatment with azacitidine (AZA) + Venetoclax (VEN).

## Case Report

**Figure 1:**
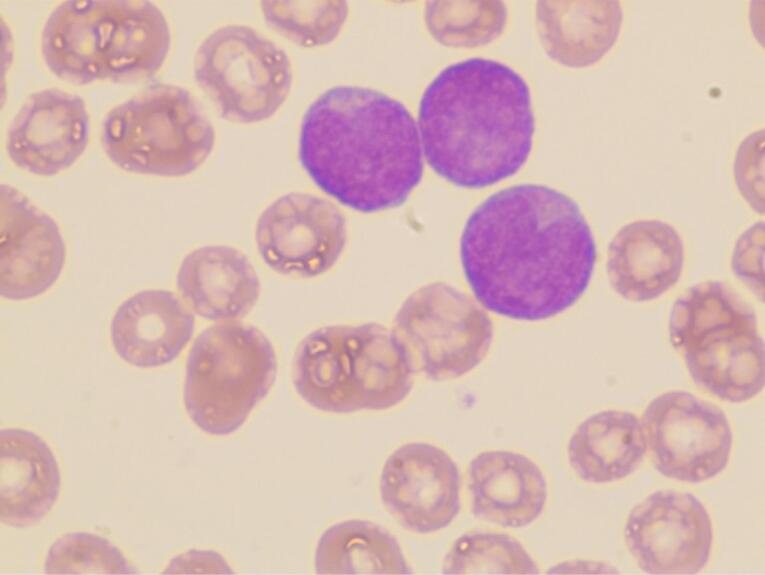
Myeloblasts in Bone Marrow, Wright-Giemsa Staining 1000 ×

 A 62-year-old male was admitted to our hospital on March 16, 2023, with a four-month history of AML and partial remission in response to therapy. The patient initially developed symptoms of chest tightness in November 2022. There were no congenital diseases, history of occupational exposure, history of viral infection, infectious diseases, residence in epidemic areas, history of drinking alcohol or smoking, and no history of special toxin exposure in the past six months. Bone marrow (BM) morphology revealed 87% myeloid blasts (Figure 1), with leukemic cells (79.2%) exhibiting positivity for CD45(dim), CD117, CD13, CD33, CD56(part), and negativity for CD34 and HLA-DR, consistent with myeloid origin. Identified mutations included *NPM1* (p.W288cfs*12, VAF 41.9%), *IDH2* (p.R140Q, VAF 45.1%), and *FLT3-ITD* (p.F594_D600dup, VAF 14.8%). Due to a mild increase in bilirubin, genetic disease gene screening by NGS revealed *UGT1A1* heterozygous variation (p.Pro364Leu), leading to the diagnosis of AML-M4 and Crigler–Najjar Syndrome. Following a two-course regimen of AZA (149 mg/dL-d7) + VEN (100 mg/d1, 200 mg/d2, 400 mg/d3‒d28) therapy, partial remission was achieved. BM suppression was reflected in the complete blood count, with a white blood cell count of 0.98 × 10^9^/L, hemoglobin level of 120 g/L, and a platelet count of 120 × 10^9^/L.

**Figure 2:**
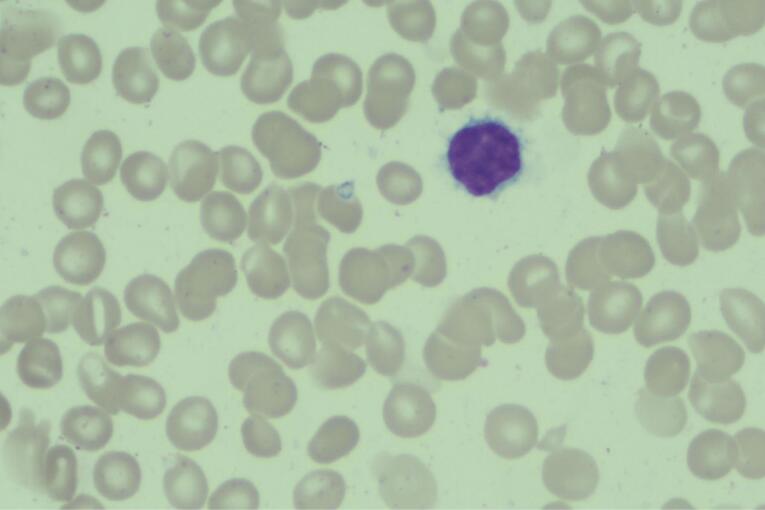
Hairy Cell in Bone Marrow, Wright-Giemsa Staining 1000 ×

 Upon transfer to our hospital, the patient underwent treatment with Aclarubicin Hydrochloride, cytarabine, G-CSF, and Gilteritinib. BM examinations on April 3, 2023, revealed significantly reduced proliferation of nuclear cells. Notably, lymphocytes constituted an unusually high proportion, accounting for 60.4%. Within this population, 6.4% were atypical lymphocytes characterized by slightly larger cell bodies, abundant gray-blue staining cytoplasm, villous-like protrusions of varying lengths, round or twisted and folded nuclei, and relatively loose chromatin (Figure 2). The granulocyte lineage comprised 34.4%, mainly consisting of granulocytes in the middle and late stages, exhibiting no apparent abnormalities. Blood film analysis displayed 1% atypical lymphocytes with the same morphological features as observed in the BM smear. Flow cytometry revealed 7.23% mature B lymphocytes in the BM sample and 12.60% in peripheral blood. These B lymphocytes were positive for CD45, CD19, CD22(bright), CD20, sIgM, CyKappa, CD103, CD123, CD25, CD11c, FMC-7(dim), HLA-DR, CD23(part), CD43(part), and negative for CD5, CD10, and CyLambda. This phenotype was consistent with HCL, with no abnormalities detected in the phenotype of immature cells in the myeloid lineage. The diagnosis of HCL was further confirmed by the presence of a BRAF mutation (p.V600E, VAF 3.75%).

 The patient underwent a combined haploidentical transplant from his son and umbilical cord blood transplant (from a male donor with HLA 10/10 match) on May 4, 2023. The transplantation followed a conditioning regimen involving total body irradiation (TBI), thiotepa, cladribine, and cytosine arabinoside. Graft-versus-host disease (GVHD) prophylaxis was administered using cyclosporin A, anti-thyroglobulin antibodies (ATG), and anti-CD25rh monoclonal antibody (MAb). Subsequently, the patient developed acute gastrointestinal GVHD, as well as acute skin and liver GVHD. These complications were effectively managed with ruxolitinib, anti-CD25rh MAb, methotrexate, and tacrolimus therapy. The patient has remained in leukemia-free remission, and as of the current date, it has been 14 months since the transplantation procedure.

## Discussion

 To the best of our knowledge, the occurrence of HCL as a novel discovery during AML treatment has not been reported previously. Concomitant AML and HCL have only been documented in two cases before.^5,6^ Previous literature reports have primarily focused on the development of AML following the treatment of HCL.^1-4^

 Classically, patients with HCL typically exhibit splenomegaly and peripheral cytopenias. In this patient’s case, laboratory examination revealed an abnormally high and increasing proportion of lymphocytes, along with a slightly enlarged spleen observed in the abdominal ultrasound examination. Notably, the proportion of atypical lymphocytes in terms of morphology was relatively low (6.4%), differing from the typical morphology associated with classical HCL cells. Fortunately, our laboratory employs an immunophenotyping panel that covers various major cell lineages. This comprehensive approach is crucial, as it significantly reduces the likelihood of overlooking HCL during diagnosis.

 We reviewed the flow cytometry data obtained at the patients’ initial diagnosis, finding that B cells accounted for 0.8% of nuclear cells. Given the absence of suspicion regarding the presence of mature monoclonal B cells, neither B cell clonality analysis nor B lymphocyte lymphoma-related antigen analysis was conducted. Consequently, it is currently challenging to definitively ascertain the existence of HCL clones at the initial diagnosis. Rimner et al and Shi et al reported cases of concomitant AML and HCL in a 58-year-old male and a 78-year-old male, respectively.^5,6^ The former received two cycles of chemotherapy including daunorubicin, cytarabine, and 2-CDA, followed by autologous stem cell transplantation. AML recurred after 1 year and 10 months.^5^ Treatment and outcomes were not specified for the latter case.^6^ Our patient underwent two courses of chemotherapy and haploidentical combined umbilical cord blood transplantation, achieving complete remission for both AML and HCL for 14 months.

 Considering the lack of previous reports on HCL in patients treated with AZA + VEN, various potential etiologies for HCL in this case are under consideration: (1) coincidental *de novo* HCL, (2) secondary to prior AML, or (3) HCL secondary to the AZA + VEN treatment. Notably, the combination of VEN with hypomethylating agents like AZA or Decitabine has shown significant improvements in the outcomes of patients newly diagnosed with AML in recent years. Determining whether AZA + VEN contributes to the development of secondary malignant disease necessitates long-term surveillance. Currently, it cannot definitively be ruled out that HCL may coexist with or be secondary to AML in our patient. HCL may precede AML, occur concurrently, or arise secondary to AML. The genetic susceptibility hypothesis may be more plausible.
